# Oral hygiene instructions and professional control as part of the treatment of desquamative gingivitis. Systematic review

**DOI:** 10.4317/medoral.22782

**Published:** 2019-03

**Authors:** María-José Garcia-Pola, Samuel Rodriguez-López, Alejandra Fernánz-Vigil, Leticia Bagán, José-Manuel Garcia-Martín

**Affiliations:** 1 MD. DDS. PhD. Professor. Section of Oral Medicine. Faculty of Medicine and Sciences of the Health. Oviedo University. Julián Clavería. 33006. Oviedo; 2 Colaborate of Oral Medicine Section. Faculty of Medicine and Sciences of the Health. Oviedo University. Julián Clavería. 33006. Oviedo. Spain; 3 Profesora Asociada de Medicina Bucal Universitat de València. Av. de Blasco Ibáñez, 15, 46010 València; 4 DDS. PhD. Assistant Professor of Oral Health & Preventive Dentistry. Faculty of Medicine and Sciences of the Health. Oviedo University. Julián Clavería. 33006. Oviedo. Spain

## Abstract

**Background:**

The aim of this present article was to evaluate the scientific evidence on the efficacy of daily hygiene and professional prophylaxis for treatment of desquamative gingivitis.

**Material and Methods:**

The present systematic review was conducted following the PRISMA protocol. Searches were carried out in Pubmed, Embase, Web of Science and Cochrane Library up to July 2018, randomized clinical trials and cohort studies on desquamative gingivitis (DG), and oral diseases joined to DG.

**Results:**

After screening, we found that nine publications met the eligibility criteria eight cohort studies and one randomized control trial. The diagnosis of the diseases corresponded to oral lichen planus (n=185), mucous membrane pemphigoid (n=13); plasma cell gingivitits (n=15) and pemphigus vulgar (n=11). The follow-up was between a week and a year after instructing patients. Dental daily hygiene and professional prophylaxis, at least with supragingival scaling and polishing have significantly improved the extension of the lesion and reduced the activity of DG, and gingival bleeding in all patients. Furthermore, these techniques have also reduced pain and gingival plaque.

**Conclusions:**

In conclusion the studies presented support the efficacy of maintaining personal and professional oral hygiene in patients with GD, reducing the clinical signs of the disease, regardless of its pathogenesis.

** Key words:**Desquamative gingivitis, oral hygiene, oral lichen planus, mucous membrane pemphigoid; pemphigus, plasma cell gingivitis.

## Introduction

In 1932, Prinz proposed the concept “desquamative gingivitis” (DG) to indicate the presence of erythema, desquamation, erosion and blisters, in marginal and attached oral mucosa (1), signs that represent different mucocutaneous disorders (2). These disorders correspond more often to oral lichen planus (OLP), pemphigoid and pemphigus, and in other cases with other immunological pathologies such as lupus erythematosus, erythema multiforme, graft versus host disease, epidermolysis bullosa acquisita, plasma cell gingivitis (PCG) and collagen diseases (3,4).

The DG appears more frequently in old women and menopause, although it can debut in young people and children. Clinically, it presents moderate pain, partly due to the deposit of plaque in gingival margin (5), being in some cases the first manifestation of the disease (6).

The study of periodontal status in patients with DG suggests that in patients with mucous membrane pemphigoid (MMP) the gingivo-periodontal status is worse than control health (7-9), in the same case, patients with OLP and pemphigus vulgaris (PV) present deeper pockets and higher loss of the clinical attachment level (10,11).

There are specific therapies that have been focused on the general manifestations of the disease linked to its pathogenesis. Topical treatment has been indicated, mainly the use of corticosteroid in different forms and prescribed with different posologies, or also systemic drugs administration such as corticosteroids, other immunosuppressants and broad-spectrum antibiotics (12,13).

As the term “desquamative gingivitis” is a clinical term that represents an oral expression of different mucocutaneous diseases, its treatment is diversified and for this reason it has expressed the importance of the control of their dental hygiene (12,13). The aim of the present systematic review is to evaluate the efficacy of individual hygiene, prophylaxis and non-surgical periodontal treatment, as a therapeutic alternative or adjuvant for the DG. The formulation of this objective refers to participants, interventions, comparisons outcomes and study design (PICOS).

## Material and Methods

Protocol and question. This study has been conducted following the PRISMA protocol system (Preferred Reporting Items for Systematic Reviews and Meta-analyses) (14), and the PICO question was formulated with the following wording: Is there an improvement in the symptoms and signs in patients with the desquamative gingivitis after oral hygiene and prophylaxsis ?

Eligibility criteria. We considered the following inclusion criteria:

Objective definition of pathology. Studies must show the objective definition of the pathology, it should correspond to the clinical term by the presence in the marginal or attached gingiva of erythema, erosion-ulceration, and accompanied or not by bullous lesions (15). The diagnosis must be by clinical, anatomopathologycally (16,17), and corroborated if were necessary with the right immunofluorescence analysis of each disease (3,15,18).

Type of the study. Design of the studies selected were of cohorts, case-control studies and randomized clinical trials focused on the importance of oral hygiene and periodontal treatment of patients with DG.

Language. The articles included should be written in English, Spanish, Portuguese or French, accompanied or not of another language.

Exclusion criteria were the following: letters, posters and conferences, narrative reviews, cases and series of cases, or studies in other languages.

Sources of information. The research was conducted up to July 8th, 2018 without limitation in the period of time of publication, through the electronic system of Pubmed, Embase, Web of Science and Cochrane Library.

Search strategy. It was carried out using the following “builder”: “desquamative gingivitis”; or “desquamative gingivitis and lichen planus”, or “desquamative gingivitis and oral lichen planus”; or “desquamative gingivitis and erythema multiforme”; or “desquamative gingivitis and mucous membrane pemphigoid”, or “desquamative gingivitis and pemphigus”, or “desquamative gingivitis and graft host” or, “desquamative gingivitis and Ig A linear”; or “desquamative gingivitis and lupus”; or “desquamative gingivitis and epidermolysis”, or “desquamative gingivitis and dermatomyositis” and “desquamative gingivitis and plasma cell”.

Study selection. Two independent authors (R-L and G-P) carried out the article selection based on inclusion criteria. In the event that there was no agreement, it would be submitted to a third reviewer (G-M).

Data collection process. After the analysis of the titles and authors, duplicated articles were discarded. Afterwards, based on the summaries the potentially relevant articles were selected. The whole texts of these were read to determine the ones that met the inclusion criteria.

Data items. We have indicated the demographic variables and type of study. The diagnosis of disease, pain intensity and signs of the disease, as well as plaque index and periodontal parameters such as bleeding index, pocket depth and attachment were noted. In addition, programs and instruction in dento-gingival hygiene and plaque control were recorded. Regarding the specific associated treatments, their guidelines, and adverse effects were also recorded as well as the time of follow-up of the patients and effects obtained.

Risk of bias in individual studies. To reduce the risk of bias, studies with a methodological design of cohort series, case-control studies and randomized control trials were selected. Also, all the patients had to be diagnosed adequately by biopsy. The results of each of the variables related to pain, signs of the disease and periodontal indexes had to be quantified in absolute values.

Summary measures. In addition to the absolute values of each measure, statistically significance through the value of “*p*” was indicated.

Risk of bias across the studies. The risk of bias were evaluated according to the Grading of Recommendations Assesment, Development and Evaluation (GRADE) which includes criteria of imprecision, inconsistency, indirectness and publication bias (19). The categories considered were high, moderate, low, and very low, in this case when true effect is likely to be substantially different from the estimate of effect.

## Results

**Figure 1:**
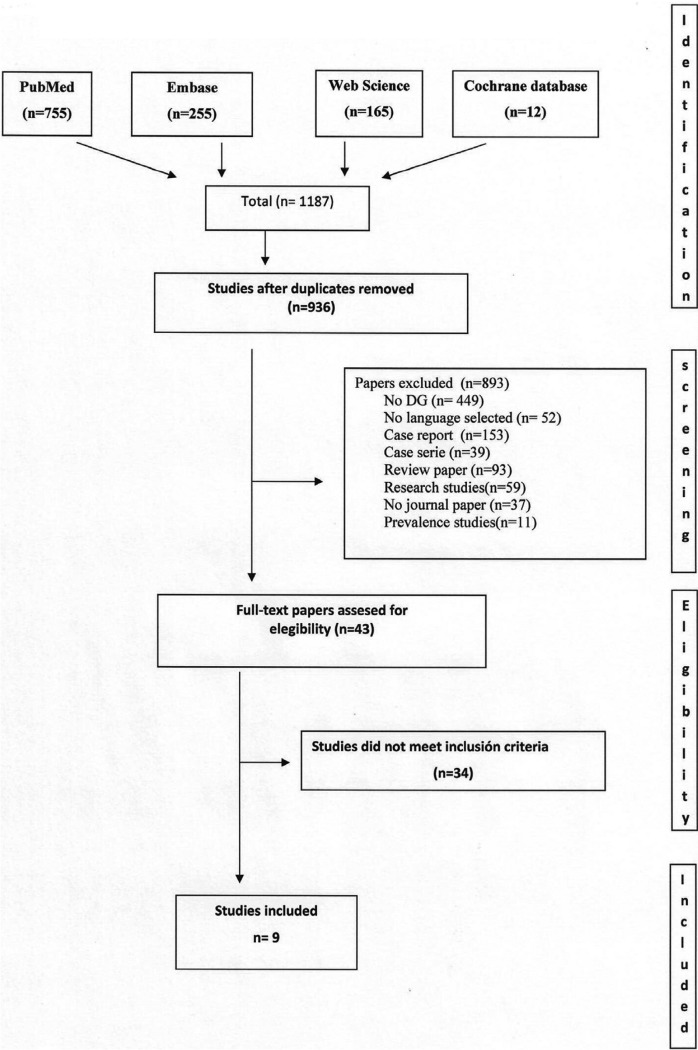
Flow diagram of data collection process according to the PRISMA statement. DG: desquamative gingivitis (14).

Study selection. Figure 1 shows the diagram of article selection (Fig. 1). After reading the eligibility paper, only nine of them were taken in the present study. The selected articles were published between 1990 and 2016, both years included (20-28), all were written in English and come from Italy (n=5), Brazil (n=1), Denmark (n=1), Spain (n=1), and The United Kingdom (n=1).

Study characteristics.  Table 1 shows the final sample of each article and demographic characteristics of patients included after the elimination of patients who did not complete the study. The total number of subjects analyzed was 224 (85.13% women). The age of patients ranged from 7 to 89 years, and the average age was 53.67 years.

**Table 1:**
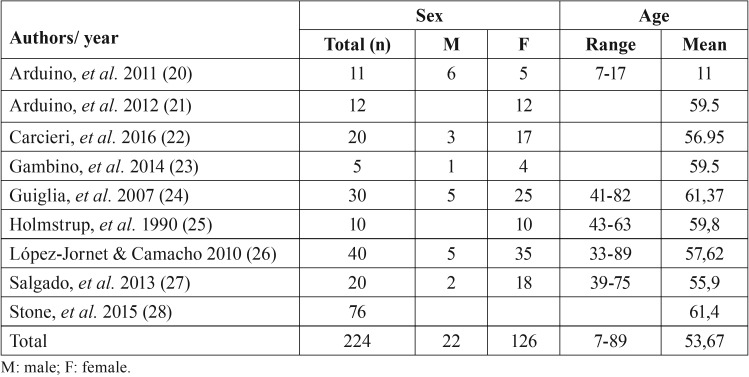
Demographic characteristics of the patients with desquamative gingivitis.

According to the methodological design, the types of studies were: randomized clinical trial (n=1) (28), and cohort (n=8) (20-27). The diagnosis of the diseases correspond to 185 OLP, 13 MMP, 15 PCG and 11 PV.

The recommended hygiene guidelines, treatments for scaling calculus, and the types of toothbrushes and interdental brushes, mouthwashes and techniques used for this purpose are indicated in  Table 2. The most commonly used brushing technique was Bass (or modified) (20-24,26), with a soft brush initially and continuing later with medium bristle brush. The mouthrinses consisted of 0.2% chlorhexidine (CHX) (20,21,23-25), twice a day, between one or two weeks and continuing with 0.12% CHX (20). Other instructions focused on the use of free lauryl sulfate toothpastes and changing the toothbrush every two weeks.

The sort of topical treatments included were the following: sodium iodine and salicylic acid, and 0.1% triamcinolone acetonide corticosteroids (26), and 0.025% of clobetasol propionate (24). Prednisone was also prescribed orally in doses of 1-1.5mg/kg/day once a day (23). No medical treatment was marked in four papers (20,21,25,27).

**Table 2:**
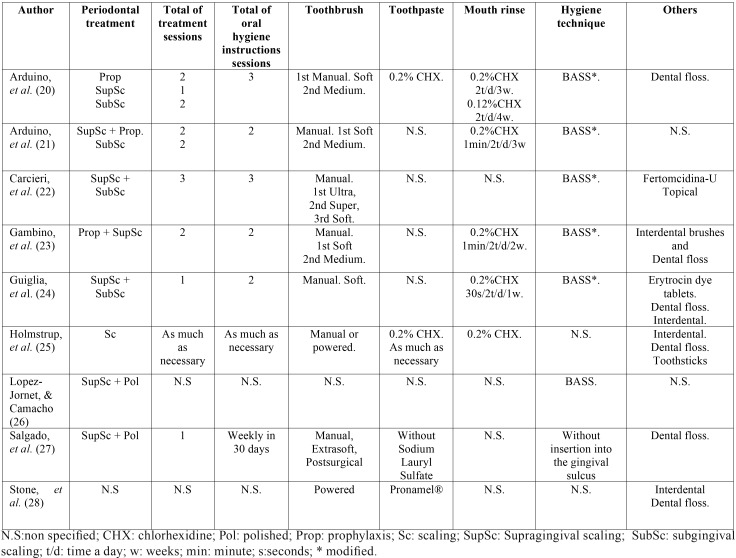
Hygiene guidelines and periodontal treatments performed.

Risk of bias within studies. There were six studies that analyzed pain levels using the visual analogue scale (VAS), and one by means of a symptom questionnaire that included spontaneous pain, eating pain, and brushing pain associated, quantified in a dichotomous way (yes or not) (25).

The extension of the lesion was collected through photography (24), and the severity of the lesion was differentiated in three degrees (based on the scale of Escudier *et al.*) (20,21), and in four grades (27,28). Another clinical parameter evaluated was the presence or absence of erosions (23).

The plaque index and other gingival and periodontal indexes are detailed in  Table 3. The number of clinic oral hygiene sessions ranged from a single session, once a week for three weeks and the possibility of insisting on each revision. Holmstrup *et al.*, considered the treatment finished when the patients were able to control their levels of plaque (25).

**Table 3:**
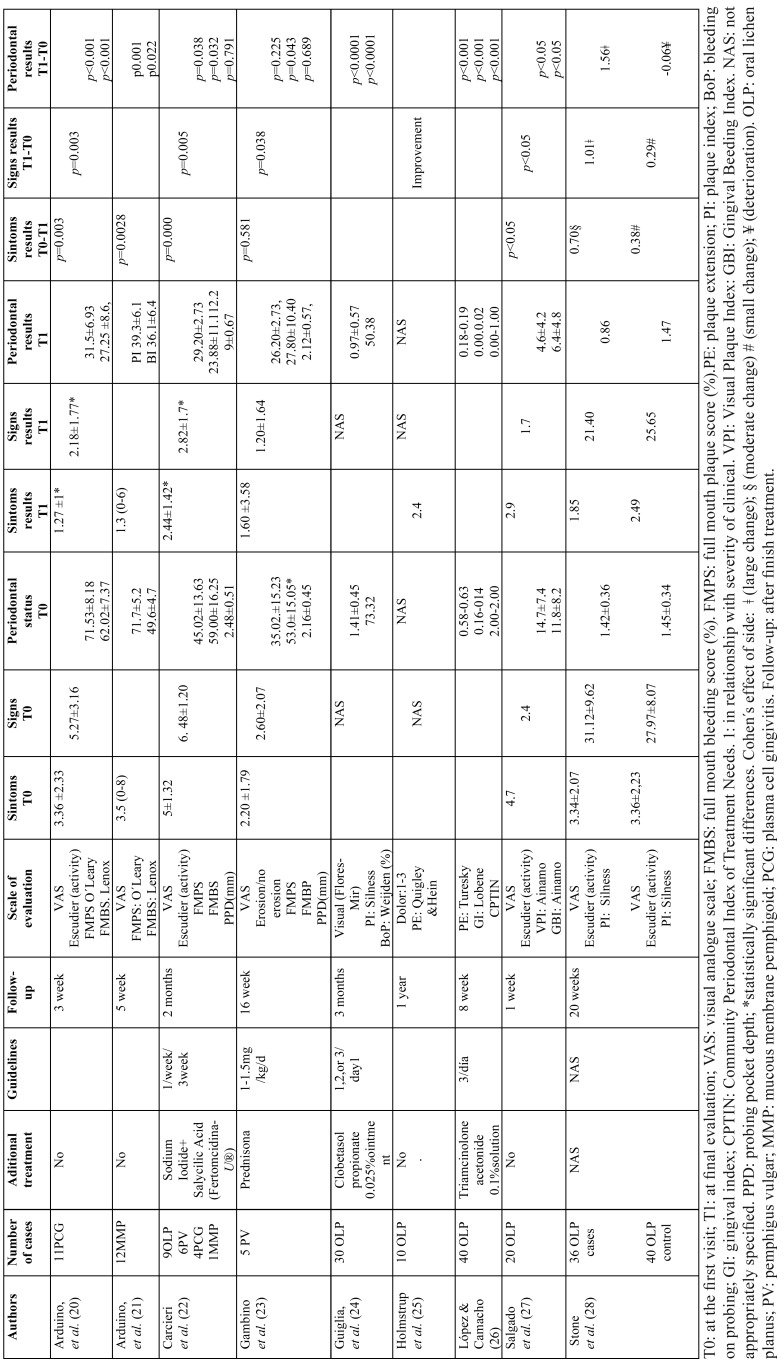
Clinical and periodontal evaluation.

The follow-up periods after patients completed their treatments range from one week (27), and one year (25).

Results of individual studies

Results of effectiveness treatment on the symptoms. The most relevant results are shown in  Table 3. The mean pain intensity according to VAS was 3.63 (with a range of 0 to 8) and its decrease was statistically significant after the week of oral hygiene intervention (27), at five weeks (21), and at two months (20,22). Otherwise, in another investigation twenty weeks after completing the learning hygiene, there was improvement in pain but no statistically significant differences in the quantification, when compared with a control group in which no intervention had been provided. Either among the patients with PV from Gambino *et al.* study (23), there was not statistically significant improvement at 16 weeks of follow-up.

Results about treatment over clinical signs. An improvement of the extension of the lesion and reduction of the activity subsequently to supragingival scaling and progress on hygienic techniques of the patients after one week (27), three weeks (20), eight weeks (22), and twenty weeks (28) was demonstrated by statistically significant differences. In addition, the performance of hygiene on the healing of PV erosions was statistically significant (23).

Results about treatment over gingival-periodontal status of patients. The hygiene techniques favored the reduction of gingival plaque in a statistically significant way in patients with PCG (20,22), PMM (21,22), and in cases of LPO (22,27,28). Other authors found statistically significant results after prophylaxis and linked to clobetasol propionate (24), or triamcinolone acetonide (26). On the contrary, there was no statistically significant improvement on plaque-control in patients diagnosed with PV (23).

The hygiene guidelines established favoured the reduction of the gingival bleeding index in all the cases tested (20-24,27). Conversely, after tartrectomy there were no favorable changes in relation to the depth of the periodontal pockets (22,23).

Risk of bias across studies. There were three studies which have shown a low quality (23,25,27), based on the GRADE checklist ( Table 4), five have shown moderate level of quality (20-22,24,26) and one high quality level (28).

**Table 4:**
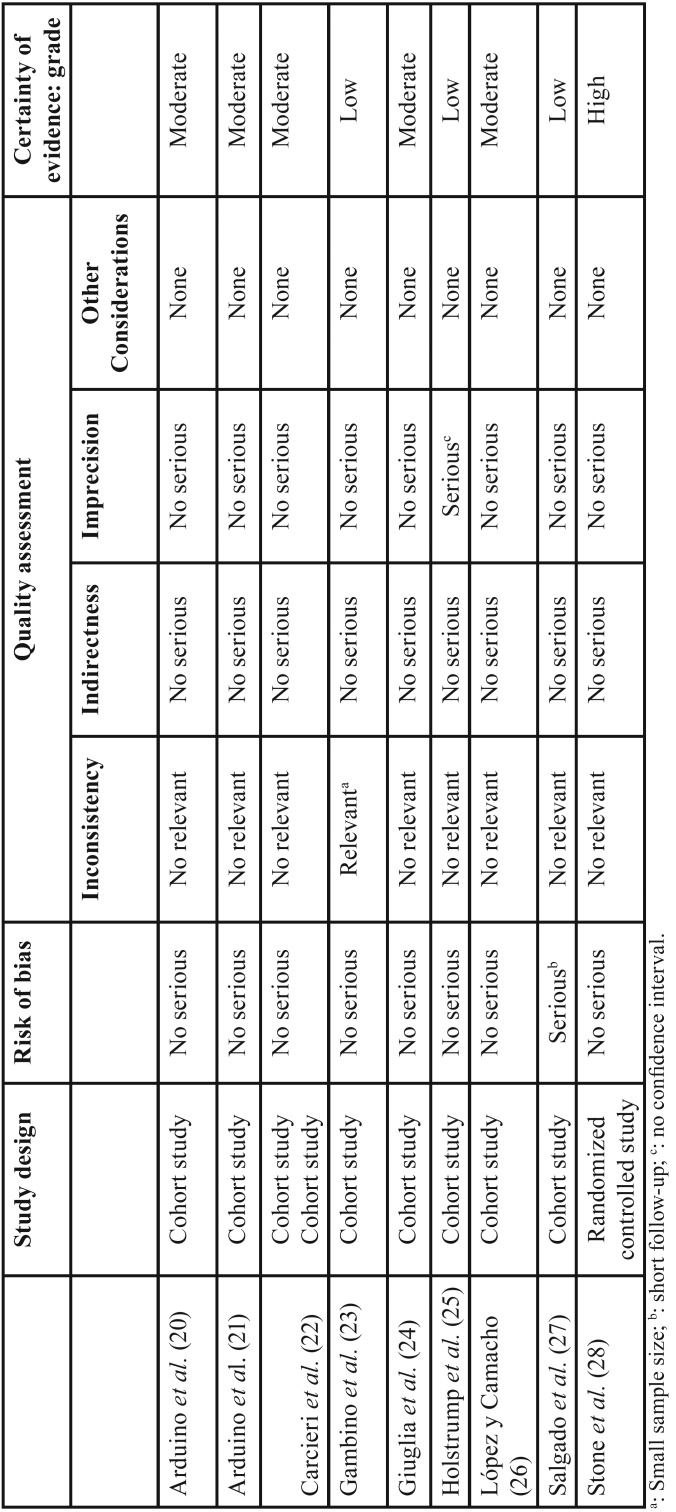
Study quality as assessed by grading of recommendations, assessment, development and evaluation (GRADE) checklist.

## Discussion

The present systematic review shows through eight cohort studies and a randomized control trial the clinical efficacy of maintaining plaque control in patients with desquamative gingivitis, instructed in keeping good hygiene as a daily habit.

DG represents a relevant oral mucosal disorder because of the diversity of its origin. Due to this fact it is not always possible to relate DG to a previous diagnosis, it is important that the oral health specialists know its concept to be able to distinguish between a classic inflammatory gingivitis and DG associated to dental plaque. The magnitude of DG is based on the high delay of its pathogenic diagnosis, approximately 83.2 days (29). Because of that the physician must identify, not only the clinical description of its erythema, erosion and/or blisters presence, but it must also be accompanied by conventional microscopic study and immunofluorescence. This fact requires the physician to perform the biopsy in the appropriate and selecting area, with the purpose of projecting the immunological diagnosis of the lesion (4).

There is a clear difference in the demographic characteristics of patients with DG. The sample studied in the present review obeys the model followed by the age patterns of DG, closed to the sixth decade, with the exception of PCG patients that debute in young people, at an average age of 11 years.

In this systematic review there were more relevant results about recovery of DG sings than pain perception. The use of VAS to evaluate pain has allowed to compare different studies, finding that pain was reduced in all cases except two of them (23,28). However, an improvement in the signs of disease activity was found in all the studies analyzed (20-22,27,28), and a reduction in the signs of erosion associated with PV (23). This would justify the applicability of the dental hygiene and dental prophilaxys in these patients with the aim of reducing the chronicity of the disease in some cases, and the use of complex treatments with systemic immunosuppressants in other cases (30).

Another important finding was the reduction of gingival bleeding and gingival plaque in all patients, although plaque reduction was not statistically significant in patients with PV (23).

The net values of these treatments range between £ 97 and £1,339. This seems to be higher than the initially proposed to the patients, however patients agree with hygiene programs and treatment protocols (31).

The main limitation of this systematic review is the heterogeneity of studies increased by the reduced number of samples; although, all the articles that we present are prospective in order to reduce the bias of the information. This heterogeneity includes patient follow-up time, which ranged from one week to one year, but it has made it possible to clarify that results can be obtained from one week after treatment. Another limitation was the inability to perform meta-analysis.

In summary, as reflected in  Table 2 and  Table 3, the studies presented support the efficacy of maintaining oral hygiene in patients with DG, reducing the clinical signs of the disease, regardless of its pathogenesis. Patients with DG should be instructed in the maintenance of proper hygiene, using different techniques, toothbrushes with soft or extra-soft bristles, which can be easily inserted over the gingival sulcus with a 45º inclination, and the use of dental floss. It is also indicated to rinse with clorhexidine twice a day, with an initial 0.2% concentration, continuing with the 0.12% concentration (one to four weeks).

## Conclusions

From this systematic review it can be concluded that the combination of appropriate gingival hygiene techniques, daily at home, in patients with DG, reinforced weekly and depending on the particular needs, the performance of periodontal treatment based on scaling and root planning (supragingival or infragingival) decrease the pain-perception, activity of the disease, dental-plaque and gingival bleeding. This technique represents a practice of first choice as a coadjuvant for the specific treatment for diseases based on DG. General dentists, periodontists and hygienists play a key role in control of DG.
